# *Trichoderma*-Induced Acidification Is an Early Trigger for Changes in *Arabidopsis* Root Growth and Determines Fungal Phytostimulation

**DOI:** 10.3389/fpls.2017.00822

**Published:** 2017-05-17

**Authors:** Ramón Pelagio-Flores, Saraí Esparza-Reynoso, Amira Garnica-Vergara, José López-Bucio, Alfredo Herrera-Estrella

**Affiliations:** ^1^Laboratorio Nacional de Genómica para la Biodiversidad-Unidad de Genómica Avanzada, Centro de Investigación y de Estudios Avanzados del IPNIrapuato, México; ^2^Instituto de Investigaciones Químico-Biológicas, Universidad Michoacana de San Nicolás de HidalgoMorelia, México

**Keywords:** plant growth, root development, symbiosis, soil pH, pH sensing, biocontrol

## Abstract

*Trichoderma* spp. are common rhizosphere inhabitants widely used as biological control agents and their role as plant growth promoting fungi has been established. Although soil pH influences several fungal and plant functional traits such as growth and nutrition, little is known about its influence in rhizospheric or mutualistic interactions. The role of pH in the *Trichoderma*–*Arabidopsi*s interaction was studied by determining primary root growth and lateral root formation, root meristem status and cell viability, quiescent center (QC) integrity, and auxin inducible gene expression. Primary root growth phenotypes in wild type seedlings and STOP1 mutants allowed identification of a putative root pH sensing pathway likely operating in plant–fungus recognition. Acidification by *Trichoderma* induced auxin redistribution within *Arabidopsis* columella root cap cells, causing root tip bending and growth inhibition. Root growth stoppage correlated with decreased cell division and with the loss of QC integrity and cell viability, which were reversed by buffering the medium. In addition, *stop1*, an *Arabidopsis* mutant sensitive to low pH, was oversensitive to *T. atroviride* primary root growth repression, providing genetic evidence that a pH root sensing mechanism reprograms root architecture during the interaction. Our results indicate that root sensing of pH mediates the interaction of *Trichoderma* with plants.

## Introduction

Plants are constantly exposed to biotic or abiotic stimuli and adjust their growth and developmental patterns to adapt and survive. Members of the fungal genus *Trichoderma* are frequently found in the rhizosphere, a narrow soil zone influenced by roots, where many species establish beneficial interactions with plants either antagonizing phytopathogens or directly influencing morphogenesis (Benítez et al., 2004; Harman et al., 2004; Harman, 2006; Druzhinina et al., 2011; Hermosa et al., 2012).

A complex chemical interaction is established between *Trichoderma* and their plant hosts comprising volatile and diffusible secondary metabolites, small peptides, and/or antibiotics, which influence root growth, branching and absorptive capacity (Samolski et al., 2012; López-Bucio et al., 2015). *T. virens* produces and releases auxinic compounds, including indole-3-ethanol (IET), indole-3-acetaldehyde (IALD), indole-3-carboxaldehyde (ICALD), and indole-3-acetic acid (IAA) (Contreras-Cornejo et al., 2009), whereas *T. atroviride* and *T. asperellum* produce the volatile 6-pentyl-2*H*-pyran-2-one (6-PP), which modulates plant growth and root system architecture (Kottb et al., 2015; Garnica-Vergara et al., 2016). *T. atroviride* also produces ethylene, and ethylene-related mutants *etr1* and *ein2* show defective root-hair induction and enhanced primary-root growth inhibition when co-cultivated with this fungus (Contreras-Cornejo et al., 2015). Thus, auxin and ethylene (ET) signaling play a major role in the *Arabidopsis* root developmental response to *Trichoderma.* Furthermore, *Trichoderma* induces plant defense responses and improves crop performance under different stress conditions (Mastouri et al., 2010, 2012; Contreras-Cornejo et al., 2011, 2014a; Salas-Marina et al., 2011; Rawat et al., 2013; Hashem et al., 2014).

The rhizosphere is the region where plant roots, soil conditions, and microorganisms interact. While *Trichoderma* root colonization is often of benefit to plants, improves nutrition, and/or enhances the degradation of toxic chemicals, the mechanisms of phytostimulation remain mostly unknown. The rhizosphere physicochemical conditions are the major driving forces influencing microbe proliferation (Husson, 2013), and no other single chemical soil characteristic is more important in determining the success of plants and soil microbes than pH (Brady and Weil, 1999). Optimum pH for growth varies considerably among plants, but most cultivated species grow well on slightly acid or neutral soils, in which root cells function properly (Marschner, 1991; Brady and Weil, 2010; Shavrukov and Hirai, 2016). However, when soil pH becomes more acid (lower than 5.5), root growth is repressed and plant yield decreases, correlating with an increase in toxic levels of aluminum (Al^3+^), manganese (Mn^2+^), iron (Fe^2+^), and protons (H^+^), as well as decrease in the availability of phosphorous (P), calcium (Ca^2+^), and magnesium (Mg^2+^) (von Uexküll and Mutert, 1995; Kochian et al., 2004; Fan et al., 2016a; Shavrukov and Hirai, 2016). An acidic pH further inhibits root cell division and elongation, and compromises meristem cell viability (Koyama et al., 1995; Yokota and Ojima, 1995; Lager et al., 2010; Graças et al., 2016).

In fungi, pH is also an important factor that affects growth, development and competition (Alkan et al., 2013). Several pathogenic fungi acidify the pH of the growth media such as *Penicillium* sp., *Botrytis cinerea*, *Sclerotinia sclerotiorum*, *Aspergillus niger*, and *Phomopsis mangiferae*; whereas *Colletotrichum* sp, *Alternaria alternata*, and *Fusarium oxysporum* alkalinize it, and this property is strongly involved in virulence regulation (Alkan et al., 2013; Prusky et al., 2016). *Trichoderma* spp. grows better in acidic conditions with an optimal growth at pH ranging from 4 to 6, and they can modify the pH of the rhizosphere (Trushina et al., 2013; Singh et al., 2014), but the consequences of fungal-mediated pH changes for root growth and development have not yet been analyzed.

Here, we hypothesized that acidification may play an important role in the configuration of root architecture and phytostimulation elicited by *Trichoderma*. Through detailed characterization of the effects of several *Trichoderma* species on *Arabidopsis* seedling growth, the fungal capacity to acidify the growth medium, the effects of low pH stress on root growth and plant development, as well as testing the responses of selected *Arabidopsis* mutants defective on pH sensing, we demonstrate the critical role of fungal acidification as an early response influencing root morphogenesis and plant growth. Moreover, since lateral root initiation started earlier or in parallel to root tip bending and root stoppage, we propose that a low pH independent program operates at the root pericycle to induce root branching.

## Materials and Methods

### Plant Material and Growth Conditions

All plants used in this study were in the *Arabidopsis thaliana* (L.) Heynh., Columbia (Col-0) background. Transgenic *Arabidopsis* lines *DR5::GFP* an auxin-inducible marker (Ottenschläger et al., 2003); *H2B::YFP* a cell viability marker (Boisnard-Lorig et al., 2001); *WOX5:GFP* a quiescent center (QC) marker in the root stem cell niche (SCN) (Sarkar et al., 2007); *CycB1:uidA* a marker of mitotic activity, expressed in the G2/M phase of the cell cycle (Colón-Carmona et al., 1999) previously characterized as well as the mutant *stop1* known to show a hypersensitive root response to low pH (salk_114108) obtained from the Nottingham Arabidopsis Stock Centre (NASC), were used for the different experiments. Seeds were surface-disinfected with 95% (v/v) ethanol for 5 min and 20% (v/v) bleach for 7 min, washed five times with distilled water, and stratified for 2 days at 4°C. Seeds were germinated and grown on agar plates containing 0.2X Murashige and Skoog (1962) medium (MS basal salts mixture, M524; PhytoTechnology), 0.6% sucrose (Sucrose: Ultrapure, MB Grade, 21938; USB Corporation) and 1% Agar (Agar, Micropropagation Grade, A111; PhytoTechnology) at pH 7. The suggested formulation is 4.3 g.L^-1^ of salts for 1x medium; we used 0.9 g.L^-1^, which we consider and refer to as 0.2X MS. For MES (2-(*N*-morpholino)ethanesulfonic acid) treatments it was included in the plant growth medium and the pH was adjusted to 7.0. All experiments were performed in an environmentally controlled growth room with a 16 h photoperiod (300 μmol m^-2^ s^-1^ of light intensity), and 22°C.

### Fungal Strain and Culture Conditions

*Trichoderma atroviride* IMI 206040 was propagated on potato dextrose agar (PDA; Difco), at 28°C for 5 days and then conidia were collected adding a small amount of sterile water into the Petri dishes and scraping the surface of the fungus. For the different experiments inoculation was carried out by placing a drop of a spore suspension containing 1 × 10^6^ spores. In the interaction assays the *Trichoderma* inoculum was placed at 5 cm from *A. thaliana* primary roots germinated and grown for 5 days on agar plates containing 0.2X MS medium. The plates, which included 10 *A. thaliana* seedlings each, were arranged in a completely randomized design. After 3 and 5 days of co-cultivation, plant growth was determined. For acidification experiments *T. atroviride* was inoculated on plates containing MS 0.2X supplied or not with bromophenol blue (0.006%) and analyzed every 24 h for 4 days.

### Analysis of Growth

Growth of primary roots was registered using a ruler. Lateral root number was determined by counting the lateral roots present in the primary root from the tip to the root/stem transition. Images were recorded using a digital camera (Nikon D3300, Osaka, Japan). The length of meristems was determined as the distance from the QC to the cell file where cells started to elongate and measured using IMAGEJ software (National Institute of Health, Bethesda, MD, United States). All experiments were repeated at least twice as indicated in the figure legends and data analyzed in the STATISTICA 10 software (Stat Soft Inc, 2011). Univariate and multivariate analyses with a Tukey’s *post hoc* test were used for testing differences in the experiments. Different letters are used to indicate means that differ significantly (*P* ≤ 0.05).

### *Trichoderma* Soluble Metabolites Experiments

*Trichoderma atroviride* was inoculated on Petri plates containing MS 0.2X covered by a sterile cellophane sheet and incubated in darkness for the indicated times in the different experiments. The cellophane was removed together with the mycelium, then *Arabidopsis* seeds or seedlings were germinated or transferred, respectively, onto the plates where *Trichoderma* was pre-grown, and further grown for the indicated times.

### Propidium Iodide Staining, GFP, and YFP Detection

For confocal microscopy, transgenic *A. thaliana* seedlings co-cultivated or not with *Trichoderma* were transferred from the growth medium to microscope slides with propidium iodide (20 μM), used as counterstain. All imaging was done using a Zeiss LSM 510 META inverted confocal microscope (Carl Zeiss, Germany) with either a 20X or 40X objective. GFP was excited with a 488 nm line of an Argon laser and propidium iodide (PI) with a 514 laser line. GFP emission was filtered with a BP 500–520 nm filter and PI emission was filtered with a LP 575 nm filter, or by using a confocal microscope (Olympus FV1000; Olympus Corp., Tokyo, Japan), with a 568-nm wavelength argon laser for excitation, and an emission window of 585–610 nm for propidium iodide and GFP or YFP fluorescence (488 nm excitation/505–550 nm emission, 514 nm excitation/527 nm emission, and 532 nm excitation/588 nm emission, respectively). Ten independent seedlings were analyzed per line, and treatment representative images were selected for figure construction.

### Histochemical Analysis of GUS Expression

Histochemical β-glucuronidase (GUS) expression was evaluated by incubating the plant tissues in 0.1% X-Gluc (5-bromo-4-chlorium-3-indolyl, β-D-glucuronide) phosphate buffer (NaH_2_PO_4_ and Na_2_HPO_4_, 0.1 M; pH 7), 10 mM EDTA, 0.1% (v/v) Triton X-100 with 2 mM potassium ferrocyanide and 2 mM potassium ferricyanide for 12 h at 37°C. Plants were cleared and fixed using the method of Malamy and Benfey (1997). For each marker line and for each treatment, at least 15 transgenic plants were analyzed.

### Determination of Developmental Stages of Lateral Root Primordia (LRP)

Lateral root primordia (LRP) were quantified 6 days after germination. Seedling roots were first cleared to enable LRP at early stages of development to be visualized and counted. Each LRP was classified according to its stage of development as reported by Malamy and Benfey (1997). The developmental stages are as follows. Stage I: LRP initiation (in the longitudinal plane, approximately eight to 10 ‘short’ pericycle cells are formed). Stage II: the LRP are divided into two layers by a periclinal division. Stage III: the outer layer of the primordium divides periclinally, generating a three-layer primordium. Stage IV: an LRP with four cell layers. Stage V: the LRP are midway through the parent cortex. Stage VI: the LRP have passed through the parent cortex layer and has penetrated the epidermis. It begins to resemble the mature root tip. Stage VII: the LRP appear to be just about to emerge from the parent root.

## Results

### Early Root Responses during *Arabidopsis* Interaction with *Trichoderma*

**FIGURE 1:**
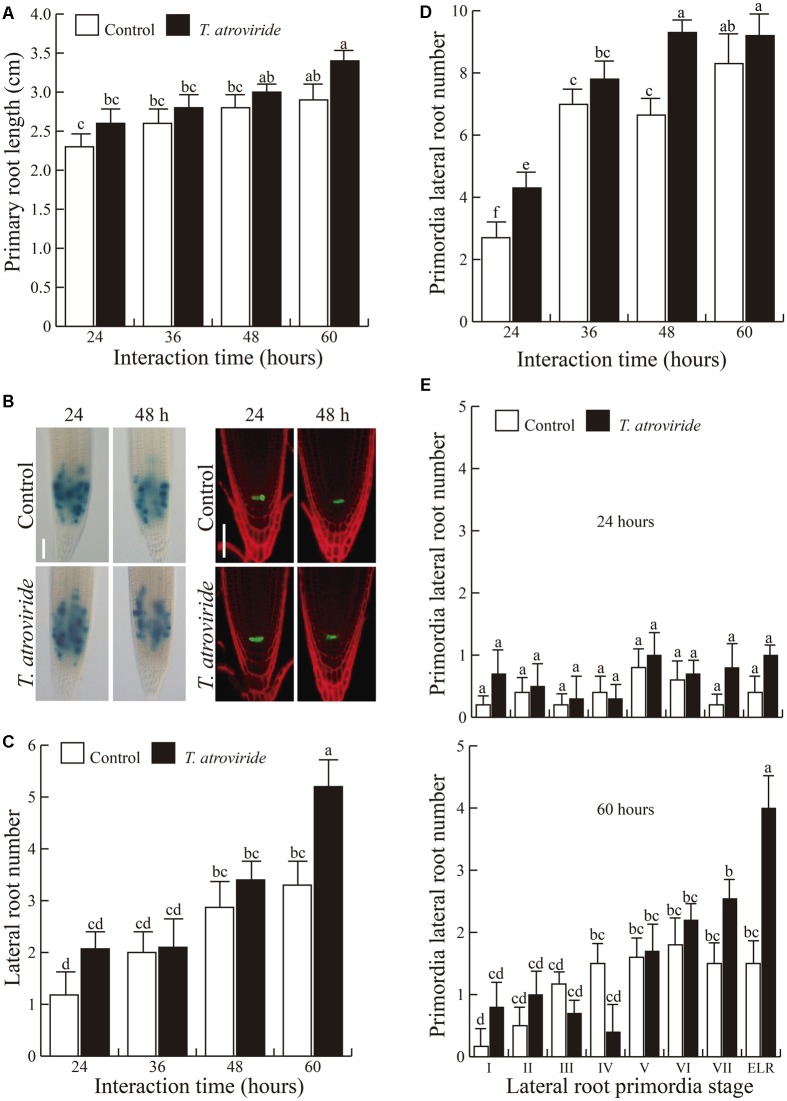
**Early responses of *Arabidopsis* to *Trichoderma atroviride***. *Arabidopsis* WT (Col-0) seeds were germinated and grown on agar solidified MS 0.2X medium. Four-day-old seedlings were inoculated with 1 × 10^6^ spores of *T. atroviride* at the opposite side of where seeds were sown and analyzed at the indicated times. **(A)** Primary root length. **(B)** *CycB1:uidA* (left) and *WOX5:GFP* (right) expression after 24 and 48 h of *Trichoderma* inoculation. **(C)** Lateral root number per plant. **(D)** Total lateral root primordia (LRP) per plant. **(E)** Number of LRP per plant after 24 and 60 h of interaction. Stage I: LRP initiation (in the longitudinal plane, approximately eight to 10 ‘short’ pericycle cells are formed). Stage II: the LRP are divided into two layers by a periclinal division. Stage III: the outer layer of the primordium divides periclinally, generating a three-layer primordium. Stage IV: an LRP with four cell layers. Stage V: the LRP are midway through the parent cortex. Stage VI: the LRP have passed through the parent cortex layer and has penetrated the epidermis. It begins to resemble the mature root tip. Stage VII: the LRP appear to be just about to emerge from the parent root. Values shown represent means with SE of at least 30 seedlings. Different letters are used to indicate means that differ significantly (*P* ≥ 0.05). Scale bars = 50 μm. The experiment was repeated three times with similar results.

To study the early responses of *Arabidopsis* seedlings to *Trichoderma*, we performed time-course experiments of *Arabidopsis* seedlings co-cultured with *T. atroviride*. We found that in the early stages of the interaction, from 24-to-60 h, primary root growth is unaffected (**Figure 1A**), and the meristem normally expresses *CyCB1:uidA*, a marker of mitotic activity, and the QC marker *WOX5:GFP*, whose corresponding WT protein is required to maintain the root SCN (**Figure 1B**). Nevertheless, *T. atroviride* clearly activated root branching at 60 h of the interaction (**Figure 1C**). The number of LRP per plant changed slightly at early stages of the interaction (24 and 48 h). However, such differences were no longer observed after 60 h (**Figure 1D**), suggesting that the differences in root branching could be due to an accelerated growth of LRP in response to *Trichoderma*. This was evidenced by analyzing the LRP developmental stages, where a decrease in the number of LRP still at the early stages of development, particularly those at stages III and IV, and an increase in the number of those more developed (stage VII) and emerged lateral roots (ELR), as early as 24 h of co-cultivation was observed (**Figure 1E**). These data suggest that *Trichoderma* increases root branching in *Arabidopsis* mainly by inducing the maturation of LRP and not *de novo* formation of LRP, as an early response that occurs independently of primary root growth inhibition.

### Late Root Responses during Interaction with *Trichoderma*

Early root responses of *Arabidopsis* to *T. atroviride* did not evidenced any negative effect. In agreement with a previous report (Contreras-Cornejo et al., 2009), we observed that *T. atroviride* promoted growth and development of lateral roots. However, after a longer time of interaction (72–96 h), a primary root growth inhibitory effect could be appreciated. Hence, we characterized in detail this late root response and its relationship with lateral root formation.

**FIGURE 2:**
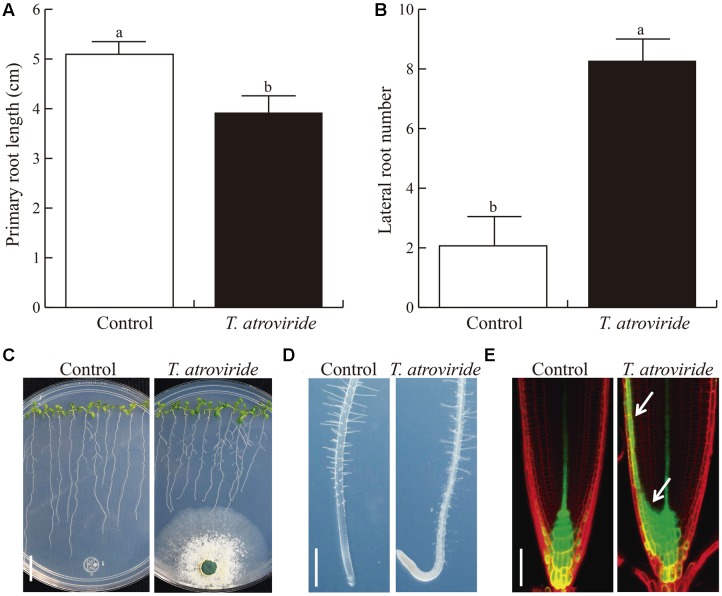
**Effects of *T. atroviride* inoculation on *Arabidopsis* root architecture. (A)** Primary root length. **(B)** Lateral root number. **(C)** Representative photographs of *Arabidopsis* seedlings co-cultivated with *Trichoderma*. **(D)** Root tips of axenic or *Trichoderma* co-cultivated WT seedlings, and *DR5::GFP* seedlings **(E)**. White arrows show auxin redistribution. *Arabidopsis* seedlings were germinated and grown for 5 days on the surface of agar plates containing MS 0.2X medium and then inoculated with *T. atroviride* at the opposite side of the plate and grown for 4 more days. Different letters are used to indicate means that differ significantly (*P* < 0.05). Error bars represents SE. Scale bars in images **(C–E)** = 1 cm, 500 and 50 μm, respectively. The experiment was repeated three times with similar results.

Inoculation with *T. atroviride* shortened primary roots (**Figures 2A,C**) while increasing lateral root number (**Figures 2B,C**). Surprisingly, the root tips bent forming a hook. The latter event is followed by inhibition of primary root elongation, stopping at the place where hook formation occurred, before contacting the mycelium (**Figures 2C,D**). Unexpectedly, upon prolonged interaction these responses were accompanied by pigmentation and chlorosis of leaves (Supplementary Figure S1). To investigate whether the bending response was specific to *T. atroviride*, we analyzed the root response to different *Trichoderma* species (*T. asperellum*, *T. koningii*, and *T. harzianum*) and about 50 other native soil isolates. Interestingly, this response was similar, regardless of the *Trichoderma* species or isolate tested (representative images are shown in Supplementary Figure S2). These results suggest that a common signal released into the growth medium may be sensed by plants, thereof triggering the observed root responses.

To understand the signaling mechanisms involved in the elicited root bending response, and since *Trichoderma* was reported to produce auxins (Contreras-Cornejo et al., 2009), we analyzed the role of auxin signaling in this process. Like in the case of gravitational stimulation, an auxin- redistribution was observed in the root curvature response to *T. atroviride*, where auxins are redistributed within the root tip and accumulate on one side of the columella root cap cells, as indicated by the expression of the auxin-induced *DR5:GFP* marker (**Figure 2E**). This redistribution of auxin likely provokes a reduction of growth on one side of the root, which in turn could lead to the formation of the hook, or causes root growth reorientation. However, the observed redistribution of auxins did not explain the subsequent root growth inhibition, suggesting the involvement of additional fungal signals in root growth inhibition.

### *T. atroviride* Has a Strong Capacity to Acidify the Growth Medium

**FIGURE 3:**
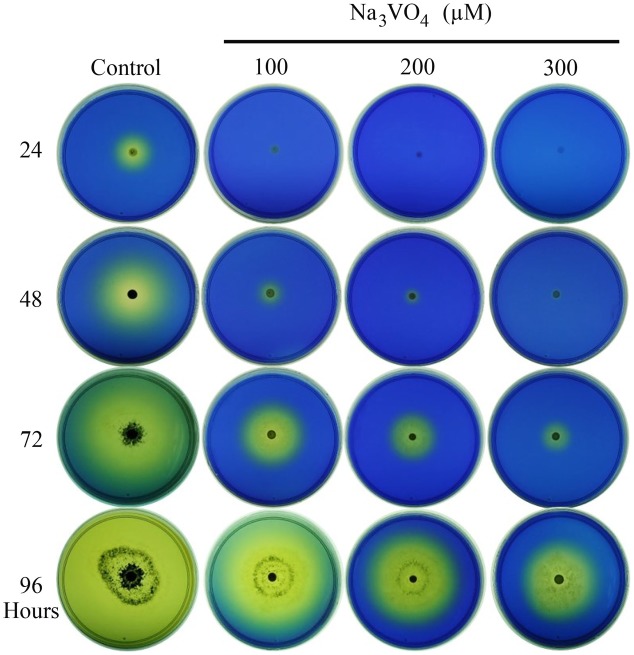
***Trichoderma* induces acidification of the medium**. *T. atroviride* was grown for the indicated times on MS 0.2X medium supplemented with bromophenol blue pH indicator and with or without vanadate at the indicated concentrations. Photographs show representative images of acidification by *Trichoderma*. The experiment was repeated twice with similar results.

Even though several studies have shown that pH is an important factor in fungal growth and development, little is known about the impact of fungal-mediated pH changes in root growth. Thus, we first determined the capacity of *T. atroviride* to modify the pH of the culture medium. For this purpose, we inoculated 1 × 10^6^ spores in MS 0.2X medium supplemented with bromophenol blue, which is used as a pH indicator. We found that *T. atroviride* strongly acidifies the medium, which occurs at least in part through proton extrusion in a process that involves vanadate sensitive ATPases, since acidification was strongly reduced by adding increasing amounts of Sodium Orthovanadate (Vanadate, Na_3_VO_4_), a competitive inhibitor of plasma membrane ATPases (**Figure 3**). Therefore, media acidification by *Trichoderma* may explain the root bending response and thus, the primary root growth inhibition of *Arabidopsis* plants co-cultivated with *T. atroviride* described above.

### Acidification Induced by *T. atroviride* Strongly Represses the *Arabidopsis* Growth and Development

**FIGURE 4:**
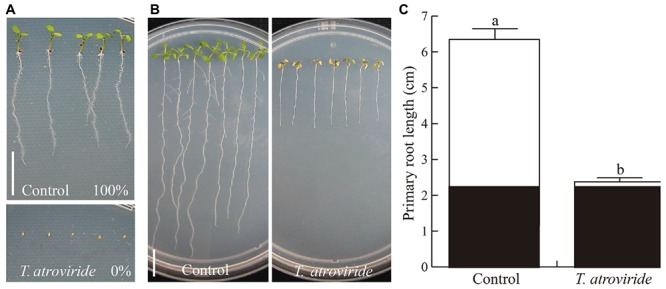
**Effects of acidification induced by *T. atroviride* on seed germination and growth of *Arabidopsis* plants. (A)** Effect on germination. **(B)** Effect on growth of *Arabidopsis* seedlings. **(C)** Primary root length. In **(A)** *Arabidopsis* (Col-0) seeds were sown on medium MS 0.2X pH 7.0 (Control) or medium where *Trichoderma* had grown for 4 days (*T. atroviride*). In **(B)** *Arabidopsis* seedlings were first grown on control medium for 4 days and then transferred to the same growth conditions used in germination assays. Black bars in graph represent the root length at the time the plant was transferred and white bars the root length 6 days after transfer. Different letters indicate means that differ significantly (*P* < 0.05). Error bars represents SE. Scale bars = 1 cm. The experiment was repeated three times with similar results.

To study the effect of *Trichoderma* induced-acidification on plant growth and development, *Arabidopsis* seeds or seedlings were sown or transferred, respectively, onto 0.2X MS growth medium where *Trichoderma* had grown. Interestingly, germination of *Arabidopsis* seeds sown on plates where *Trichoderma* had grown for 96 h was completely inhibited, in contrast with germination on control plates where all seeds germinated and seedlings developed normally (**Figure 4A**). Similar results were observed in *Arabidopsis* seedlings in transfer assays in which 4 days old *Arabidopsis* seedlings grown on MS 0.2X were transferred to control media or media where *T. atroviride* had been grown, and allowed to grow for 6 additional days. In this case, we observed that *Arabidopsis* primary root growth and overall plant development were inhibited in *Trichoderma*-treated media, as compared with the continuing growth observed for control plants (**Figures 4B,C**). Moreover, when the experiment was repeated allowing *Trichoderma* to grow on a cellophane sheet for 18, 24, 30, or 36 h, we observed a gradual plant response. Evident inhibition was observed when plants were transferred onto media where *Trichoderma* had grown for 30 h, and growth was completely inhibited six hours later (Supplementary Figure S3A). Similarly, *Arabidopsis* seed germination occurred when sown on media were *Trichoderma* had been grown for 30 h but growth stopped almost immediately, and no germination was observed by 36 h (Supplementary Figure S3B).

To determine if plant growth repression was indeed due to changes in the media conditions provoked by *Trichoderma*, we performed another experiment, in which *T. atroviride* was grown on un-buffered MS (0.2X) medium with initial pH 7 on a side of the plates for 48 h, time in which the growth medium has not been completely acidified by *Trichoderma*, and then *Arabidopsis* seeds were germinated and allowed to grow on the opposite side of the Petri dish. Interestingly, in seedlings that were grown under this condition, root growth orientation was affected, avoiding the area influenced by *Trichoderma*, compared with the normal vertical root growth of untreated seedlings (Supplementary Figure S3C). These results indicate that plants may sense the gradual changes in pH and adjust their root growth to escape from strongly acidic conditions.

### The Growth Repressing Effects of *Trichoderma* on *Arabidopsis* are Associated with Acidification

**FIGURE 5:**
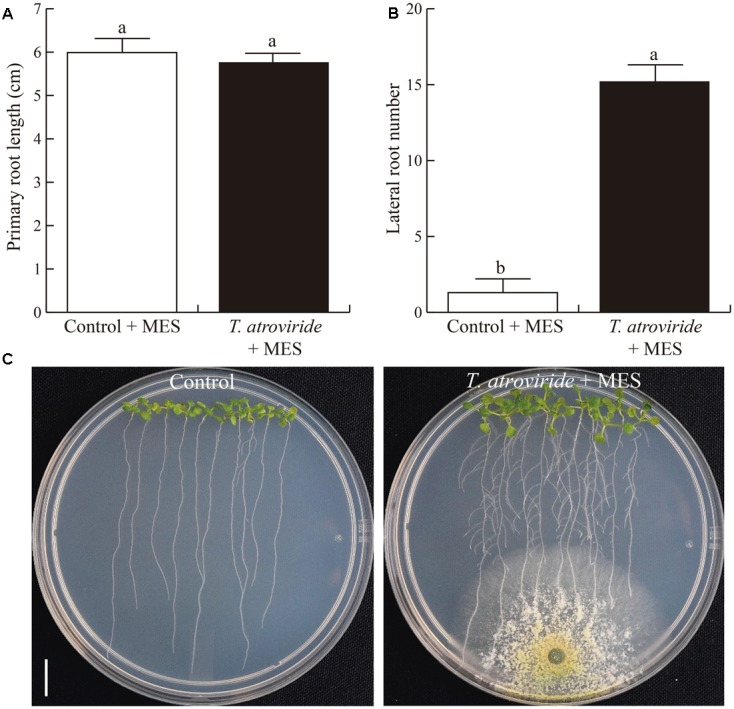
**Effects of *T. atroviride* inoculation on *Arabidopsis* growth under buffered medium. (A)** Primary root length. **(B)** Lateral root number. **(C)** Representative photographs of *Arabidopsis* seedlings co-cultivated with *Trichoderma*. *Arabidopsis* seedlings were germinated and grown on MS 0.2X medium buffered with MES 0.12%, after 5 days *T. atroviride* was inoculated at the opposite side of the plate and grown for 4 additional days. Different letters are used to indicate means that differ significantly (*P* < 0.05). Error bars represents SE. Scale bar = 1 cm. The experiment was repeated three times with similar results.

**FIGURE 6:**
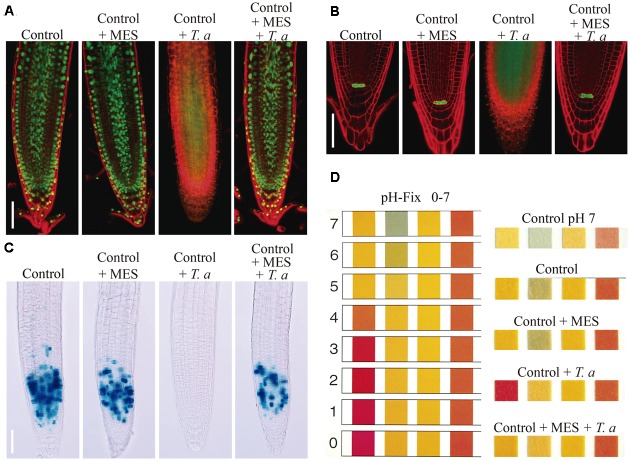
**Acidification induced by *T. atroviride* impairs the *Arabidopsis* root meristem functionality. (A)** Expression of vital marker *H2B::YFP*. **(B)** Expression of the root quiescent center (QC) marker *WOX5:GFP*. **(C)** Expression of the cell division marker *CycB1:uidA*. **(D)** Estimated pH on the different growth conditions. Five-day-old seedlings of different transgenic lines used were transferred to the indicated treatments and analyzed 24 h later. Photographs show representative images of at least 10 seedlings analyzed per experiment. Scale bars = 50 μm. These experiments were repeated twice with similar results.

All our findings on the root response of *Arabidopsis* to *Trichoderma*, were tightly correlated with the reported effects of low pH on plants (Koyama et al., 2001; Kang et al., 2013; Kobayashi et al., 2013). Thus, we evaluated the growth of plants in interaction with *Trichoderma* in pH-buffered media. Under these conditions, primary root growth of *Arabidopsis* seedlings co-cultivated with *Trichoderma* was not inhibited at all, as compared to control plants without *Trichoderma* (**Figure 5A**). Moreover, lateral root emergence was strongly stimulated, with 7–8-fold more lateral roots than in plants without *Trichoderma*, which were also much longer than those in the control (**Figures 5B,C**). Similar results were obtained in experiments in which *Arabidopsis* seeds were directly germinated (Supplementary Figure S4) or seedlings transferred (Supplementary Figure S5) to buffered medium (pH 7) where *Trichoderma* had been grown. Thus, buffering the medium eliminated the negative effects caused by *Trichoderma* on un-buffered media. In addition, we tested the toxic effects of low pH provoked by *T. atroviride* on *Arabidopsis* primary roots, to determine if it could be responsible for the observed growth inhibition. For this purpose, we analyzed cell viability by monitoring the expression of the *H2B::YFP* reporter construct (**Figure 6A**), which is specifically expressed in the nuclei of living cells (Boisnard-Lorig et al., 2001), and by using a vital staining with propidium iodide, by confocal microscopy. Further, as root growth is maintained by the SCN, which includes cells of the QC in the root apical meristem (van den Berg et al., 1997; Bennett and Scheres, 2010), we also followed the status of the QC by monitoring the expression of the reporter construct *WOX5:GFP* (**Figure 6B**). Finally, we analyzed the expression of the cell division marker *CyCB1:uidA* (**Figure 6C**), which is expressed only in cells in the G2/M transition of the cell cycle in the primary root meristem (Colón-Carmona et al., 1999). In all cases, the expression of each reporter construct was lost in the primary root meristem of plants grown on un-buffered medium in the presence of *Trichoderma*, correlating with primary root growth inhibition (**Figures 6A–C**) and a clear pH drop (**Figure 6D**). Together, these data indicate that media acidification by *T. atroviride* strongly affects cell viability and cell division in primary roots, consequently impairing meristem functionality.

### *Stop1* Response to *T. atroviride* Supports the Role of pH in the Plant–*Trichoderma* Communication

**FIGURE 7:**
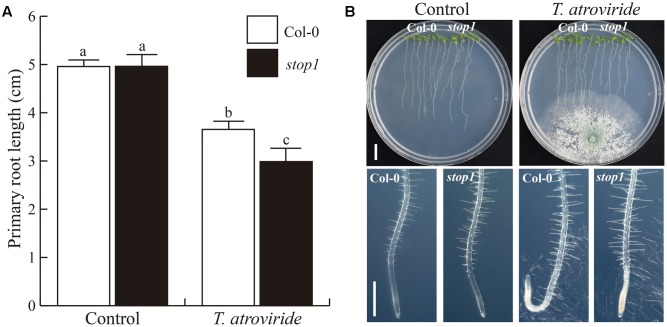
**Effect of *T. atroviride* on growth of *Arabidopsis* WT and *stop1* mutants. (A)** Six-day-old *A. thaliana* WT seedlings and *stop1* mutant were co-cultivated with 1 × 10^6^ spores of *T. atroviride*, by placing the fungus on the opposite side of the Petri plate, where seeds were sown. **(A)** Effect on primary root length. **(B)** Photographs of WT and *stop1* seedlings. Different letters indicate means statistically different at *P* < 0.05. Error bars represents SE. Scale bars = 1 cm and 500 μm. This experiment was repeated three times with similar results.

**FIGURE 8:**
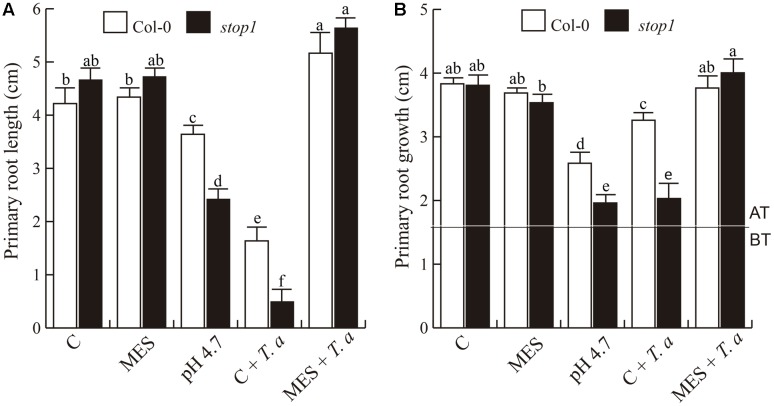
**Sensitivity of *stop1* to acidification induced by *T. atroviride*. (A,B)** Primary root length. **(A)** Seeds were germinated and grown directly under the indicated conditions or **(B)** on a pH 7.0 medium for 4 days and then transferred to the indicated treatments and analyzed 3 days after transfer. BT and AT in graph **(B)** indicate the root growth before and after transfer, respectively. Different letters in graphic indicate means statistically different at *P* < 0.05. This experiment was repeated twice with similar results.

The *Arabidopsis* mutant *stop 1* (*sensitive to proton rhizotoxicity 1*) is well-known to be hypersensitive to low pH (Iuchi et al., 2007; Sawaki et al., 2009). Therefore, we evaluated whether *stop1* was also oversensitive to *T. atroviride* or not. Interestingly, in the presence of *Trichoderma*, the primary root of the *stop1* mutant stopped growing much earlier than WT *Arabidopsis* seedlings (**Figures 7A,B**), but root bending was not observed (**Figure 7B**). It is likely that the root tip of the *stop1* mutant could not bend or form a hook, because of its greater sensitivity to low pH. Indeed, the primary root tip of the mutant showed clear signs of deterioration. This sensitivity was more clearly confirmed in two independent experiments in which *T. atroviride* had been grown for 27 h on the plant growth media supplied or not with MES buffer (**Figure 8**). As shown in **Figure 8**, primary root growth of the *stop1* mutant was significantly reduced both at 10-dag (**Figure 8A**) and 3-days after transfer (dat) (**Figure 8B**). In both cases exposure to *T. atroviride* resulted in primary root growth similar to that observed at pH 4.7. In addition, we determined the effects of these treatments on cell division and elongation by measuring the primary root meristem size and length of fully developed cortical cells at the differentiation zone of 5 days old WT and *stop1 Arabidopsis* seedlings 48 h after transfer. Strong primary root growth inhibition of WT and *stop1* seedlings under low pH (4.7) and *T. atroviride* treatment correlated with smaller cortical cells and a smaller primary root meristem (Supplementary Figure S7). None of the negative effects caused by *Trichoderma* on the *stop1* mutant on un-buffered media was observed when media was buffered with MES (**Figure 8** and Supplementary Figure S6). The sensitivity of the *stop1* mutant to *Trichoderma* indicates that the transcription factor STOP1 is involved in mediating the *Arabidopsis* root responses to media acidification by *T. atroviride.*

## Discussion

The beneficial effects of *Trichoderma* on plants such as stimulation of growth, nutrient uptake, induction of defense responses, and indirectly due to its mycoparasitic activity have been widely documented (Altomare et al., 1999; Benítez et al., 2004; Harman et al., 2004; Shoresh et al., 2010; Contreras-Cornejo et al., 2013; López-Bucio et al., 2015). In this study, we show that *Arabidopsis* growth promotion is clearly observed during the early stages of the interaction and that acidification by *Trichoderma* plays an essential role in the *Trichoderma*-plant interaction.

Growth promotion effects of *Trichoderma*, reflected in root branching patterns and its correlation with plant biomass production have been studied to some extent but are not yet well understood. The participation of auxins in modulating these effects is supported by the induction of the expression of the auxin-inducible marker gene *DR5:uidA* in roots and shoots of WT plants and by the reduced response of *Arabidopsis* mutants affected in auxin transport or signaling to *Trichoderma virens* (Contreras-Cornejo et al., 2009). Although *Trichoderma* produces auxins and other diffusible compounds, which may modulate plant growth and development, the whole phytostimulation program may not solely or completely be explained by an auxinic mechanism, since IAA production is strain dependent and can be affected by diverse external stimuli (Nieto-Jacobo et al., 2017). Furthermore, some *Trichoderma* strains inhibit the auxinic root response of *Arabidopsis* primary roots (Nieto-Jacobo et al., 2017). In this regard, recent findings suggest that growth promotion induced by *Trichoderma* during the early plant–*Trichoderma* interaction stages could be attributed mostly to volatile organic compounds (VOCs) more than to the release of auxins or other diffusible compounds by the fungus. Exposure of plants to VOCs of different *Trichoderma* species have been found to stimulate plant growth, chlorophyll content, and plant size and biomass, which could be correlated with an enhanced soil exploratory capacity, better rooting and an enhanced capacity to take up nutrients and water (Hung et al., 2013; Contreras-Cornejo et al., 2014b; Lee et al., 2016; Nieto-Jacobo et al., 2017). It is noteworthy, that we found that longer interaction times with *Trichoderma* had clear detrimental effects on primary roots compared with untreated plants, such as root tip bending and growth inhibition, which correlate with anthocyanin-like pigmentation of leaves and the eventual development of leaf chlorosis. Anthocyanin production in leaves has been found to occur as consequence or parallel effect of plant defense induction in response to *Trichoderma* (Contreras-Cornejo et al., 2011). Based on our findings, we suggest that anthocyanin accumulation could be related with a plant response to acidification by *Trichoderma* rather than to the induction of the plant defense response.

Rhizosphere acidification by *Trichoderma* spp. particularly *Trichoderma harzianum* strain T-22, has been reported in a couple of studies, one of them performed by Altomare et al. (1999), which investigated the fungal capacity to solubilize, *in vitro*, insoluble or sparingly soluble minerals by acidification of the medium. In their report the authors indicated that *Trichoderma* acidified the medium, but concluded that acidification was not the major mechanism of solubilization of insoluble minerals. Similarly, Sofo et al. (2012) found that *T. harzianum* T22 acidified the growth medium and that this may account for its beneficial effects on plants under hostile growth conditions. More recently, medium acidification by *T. atroviride* and *T. virens* was also observed in co-cultivation experiments with *Arabidopsis*, a phenomenon that was correlated with the plant promoting effects exerted by *Trichoderma* on *Arabidopsis* seedlings (Contreras-Cornejo et al., 2016).

It is known that plants can naturally acidify the rhizosphere to improve nutrient availability or uptake, and this may be particularly relevant in alkaline calcareous soils in which phosphate and iron are very limiting, but only a slight acidification can be beneficial for plant growth and development in soils with neutral or slightly acid pHs (Marschner, 1991; Hinsinger et al., 2003), while strong acidification negatively affects plant growth. Here, we found that *T. atroviride* strongly acidified the medium, through a process mediated at least in part by H^+^-ATPases, because the addition of sodium orthovanadate (Na_3_VO_4_), an ATPase inhibitor reduced acidification in a dose-dependent manner. Our data indicate that media acidification by *Trichoderma* has a clear detrimental effect on *Arabidopsis*, affecting plant development, from seed germination to root and shoot growth. These findings are consistent with the effects of low pH reported in various studies, where acidity was found to affect *Arabidopsis* root growth (Koyama et al., 1995, 2001; Kang et al., 2013), and inhibit seed germination of different plants (Fan and Li, 1999; Zeng et al., 2005). Thus, the inhibitory effects observed on *Arabidopsis* seedlings in co-cultivation with *T. atroviride* may be explained by a high level of acidification. Furthermore, when the *Arabidopsis*-*Trichoderma* experiments were carried out under buffered conditions, a clear increase in growth promotion by *Trichoderma* was observed, and the negative effects (i.e., germination and growth inhibition) were negligible, restoring completely plant growth. These results correlated with the expression of different markers used to test root meristem functionality as well as with pH levels observed under the different growth conditions. Similar detrimental effects on *Arabidopsis* root growth were recently reported for certain *Trichoderma* strains (Nieto-Jacobo et al., 2017). Although, the authors associated this response with an impaired auxin signaling, in our view, root growth repression may rather be a consequence of loss of root meristem functionality caused by acidification.

Low pH stress is related with Al toxicity, because at low pH the highly toxic Al^3+^ severely affects plant growth. However, increasing evidence supports that toxic H^+^ and Al^3+^ elicit different adaptive mechanisms in plants (Shavrukov and Hirai, 2016). In bacteria and fungi, different pH-signaling pathways have been identified (Arst and Peñalva, 2003; Yuan et al., 2008), but in plants no specific mechanisms for pH sensing have been reported. The transcription factor *STOP1* is one of the very few regulators of gene expression in plant responses to Al^3+^ and low pH stress (Iuchi et al., 2007; Sawaki et al., 2009). Accordingly, we found that the *Arabidopsis* mutant *stop1* showed higher root growth repression when co-cultivated with *Trichoderma* than WT seedlings, evidenced by a shorter primary root and absence of root bending or hook in the root tip. Such oversensitivity was confirmed in experiments where the WT and the *stop1* mutants were grown in medium supplemented with MES buffer, in which *Trichoderma* had been grown, supporting a role of pH as a signal in the interaction of *Trichoderma* with plants. Taken together, these results suggest that the pH status could be sensed by roots to activate a signaling cascade that modulates root growth and its orientation, and possibly, to activate lateral root initiation.

A recent report demonstrated that the inhibitory effects of low pH on root growth are mediated by an adaptive plant response rather than by a direct toxic effect of H^+^, because direct root exposure to low pH, suppressed root growth and caused high cell death, while roots exposed gradually to the low pH stress stopped growth but maintained cell viability (Graças et al., 2016). Although the molecular components mediating the root responses to pH or their possible link with auxin signaling are currently unknown, the idea of pH as a signal in plant–microbe interactions is supported by global gene expression analyses, which showed that low pH alters the expression of genes related to auxin signaling, pathogen elicitors, and defense-associated hormones. It is possible that pH-sensing and Ca^2+^ signaling may be mediated by proton effects on inward rectifying K^+-^channels or via a pH specific sensor (Lager et al., 2010). The fact that the *Arabidopsis aux1-7* mutant is hypersensitive to low pH, suggests an important role of auxin transport in root growth response to acidification (Inoue et al., 2016), and since the same mutant had a reduced response to *T. virens* inoculation in terms of shoot biomass production and lateral root development (Contreras-Cornejo et al., 2009), we propose that normal auxin transport is important for plant growth and root adaptation to low pH following *Trichoderma* inoculation. In addition, the nitrate transporters *NRT1.1* and *OsNRT2.3b* are involved in H^+^ resistance in *Arabidopsis* and rice, respectively, a phenomenon that depends on their nitrate uptake activity (Fang et al., 2016; Fan et al., 2016b). The relationship of *Trichoderma* with nitrate nutrition of plants represents an interesting research avenue to follow.

## Conclusion

**FIGURE 9:**
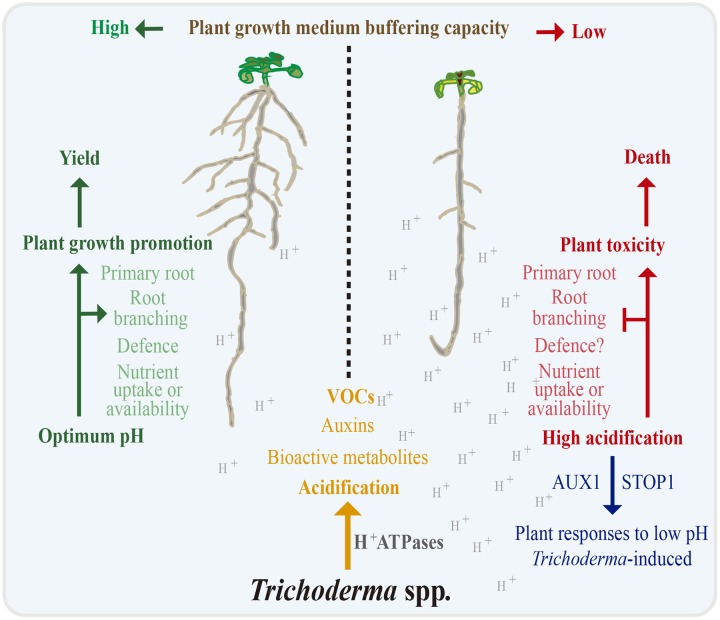
**Model for the interaction of *Trichoderma* with plants**. Rhizosphere acidification by *Trichoderma* together with auxins, VOCs, and other bioactive molecules reconfigure root architecture and may promote or impair plant growth depending upon soil pH conditions. STOP1 and AUX1 likely act in a root pH sensing pathway to adapt the root system to the acidification likely via lateral root production.

This report provides compelling evidence that root sensing of pH mediates the interaction of *Trichoderma* with plants. Rhizosphere acidification by *Trichoderma* may influence the root developmental response to auxins, VOCs, and other bioactive molecules and stimulate or repress different plant processes in a plant specific manner and depending upon the soil characteristics (**Figure 9**). We have further identified STOP1 as a critical factor in mediating root adaptation to fungal-induced acidification, which might act in a pathway involving auxin signaling and transport, where AUX1 links acidity to growth responses and plant adaptation to H^+^ stress. These data may help in the management of *Trichoderma* based strategies for crop improvement, and may aid in the identification of more efficient *Trichoderma*-strains for their use in bio-fertilizers or bio-inoculant formulation to increase plant growth and yield.

## Author Contributions

RP-F designed and performed experiments, collected, and interpreted data; AG-V performed experiments; SE-R performed experiments and provided technical support; JL-B and AH-E: designed experiments and contributed to data interpretation. RP-F, JL-B, and AH-E wrote the manuscript.

## Conflict of Interest Statement

The authors declare that the research was conducted in the absence of any commercial or financial relationships that could be construed as a potential conflict of interest.

## References

[B1] Alkan N. Espeso E. A. Prusky D. (2013). Virulence regulation of phytopathogenic fungi by pH. *Antioxid. Redox Signal.* 19 1012–1025. 10.1089/ars.2012.5062 23249178

[B2] Altomare C. Norvell W. A. Bjorkman T. Harman G. E. (1999). Solubilization of phosphates and micronutrients by the plant-growth-promoting and biocontrol fungus *Trichoderma harzianum* Rifai 1295-22. *Appl. Environ. Microbiol.* 65 2926–2933. 10388685 10.1128/aem.65.7.2926-2933.1999 PMC91438

[B3] Arst H. N. Peñalva M. A. (2003). pH regulation in *Aspergillus* and parallels with higher eukaryotic regulatory systems. *Trends Genet.* 19 224–231. 10.1016/S0168-9525(03)00052-0 12683976

[B4] Benítez T. Rincón A. M. Limón M. C. Codón A. C. (2004). Biocontrol mechanisms of *Trichoderma* strains. *Int. Microbiol.* 7 249–260. 15666245

[B5] Bennett T. Scheres B. (2010). Root development-two meristems for the price of one? *Curr. Top. Dev. Biol.* 91 67–102. 10.1016/S0070-2153(10)91003-X 20705179

[B6] Boisnard-Lorig C. Colon-Carmona A. Bauch M. Hodge S. Doerner P. Bancharel E. (2001). Dynamic analyses of the expression of the HISTONE:YFP fusion protein in *Arabidopsis* show that syncytial endosperm is divided in mitotic domains. *Plant Cell.* 13 495–509. 10.1105/tpc.13.3.495 11251092 PMC135513

[B7] Brady N. C. Weil R. R. (1999). *In “Nature and Properties of Soils.”* Upper Saddle River, NJ: Prentice-Hall.

[B8] Brady N. C. Weil R. R. (2010). *Elements of the Nature and Properties of Soils.* Hoboen, NJ: Pearson Education International.

[B9] Colón-Carmona A. You R. Haimovitch-Gal T. Doermer P. (1999). Spatiotemporal analysis of mitotic activity with a labile cyclin-GUS fusion protein. *Plant J.* 20 503–508. 10.1046/j.1365-313x.1999.00620.x 10607302

[B10] Contreras-Cornejo H. A. López-Bucio J. S. Méndez-Bravo A. Macías-Rodríguez L. Ramos-Vega M. Guevara-García A. (2015). Mitogen-activated protein kinase 6 and ethylene and auxin signaling pathways are involved in *Arabidopsis* root-system architecture alterations by *Trichoderma* atroviride. *Mol. Plant-Microbe Interact.* 28 701–710. 10.1094/MPMI-01-15-0005-R 26067203

[B11] Contreras-Cornejo H. A. Macías-Rodríguez L. I. Alfaro-Cuevas R. López-Bucio J. (2014a). *Trichoderma* spp. improve growth of *Arabidopsis* seedlings under salt stress through enhanced root development, osmolite production, and Na+ elimination through root exudates. *Mol. Plant-Microbe Interact.* 27 503–514. 10.1094/MPMI-09-13-0265-R 24502519

[B12] Contreras-Cornejo H. A. Macías-Rodríguez L. Beltrán-Peña E. Herrera-Estrella A. López-Bucio J. (2011). *Trichoderma*-induced plant immunity likely involves both hormonal and camalexin-dependent mechanisms in *Arabidopsis thaliana* and confers resistance against necrotrophic fungi *Botrytis cinerea*. *Plant Signal. Behav.* 6 1554–1563. 10.4161/psb.6.10.17443 21931272 PMC3256384

[B13] Contreras-Cornejo H. A. Macias-Rodriguez L. del-Val E. Larsen J. (2016). Ecological functions of *Trichoderma* spp. and their secondary metabolites in the rhizosphere: interactions with plants. *FEMS Microbiol. Ecol.* 92:fiw036 10.1093/femsec/fiw036 26906097

[B14] Contreras-Cornejo H. A. Macías-Rodríguez L. I. Cortés-Penagos C. López-Bucio J. (2009). *Trichoderma virens*, a plant beneficial fungus, enhances biomass production and promotes lateral root growth through an auxin-dependent mechanism in *Arabidopsis*. *Plant Physiol.* 149 1579–1592. 10.1104/pp.108.130369 19176721 PMC2649400

[B15] Contreras-Cornejo H. A. Macías-Rodríguez L. I. Herrera-Estrella A. López-Bucio J. (2014b). The 4-phosphopantetheinyl transferase of *Trichoderma virens* plays a role in plant protection against *Botrytis cinerea* through volatile organic compound emission. *Plant Soil.* 379 261–274. 10.1007/s11104-014-2069-x

[B16] Contreras-Cornejo H. A. Ortiz-Castro R. López-Bucio J. (2013). “Promotion of plant growth and the induction of systemic defence by *Trichoderma*: physiology, genetics and gene expression,” in *Trichoderma: Biology and Applications*, eds Mukherjee P. K. Horwitz B. A. Singh U. S. Mukherjee M. Schmoll M. (Walingford: CABI), 173–194.

[B17] Druzhinina I. S. Seidl-Seiboth V. Herrera-Estrella A. Horwitz B. A. Kenerley C. M. Monte E. (2011). *Trichoderma*: the genomics of opportunistic success. *Nat. Rev. Microbiol.* 9 749–759. 10.1038/nrmicro2637 21921934

[B18] Fan H. B. Li C. R. (1999). Effects of simulated acid rain on seedling emergence and growth of five broad-leaved species. *J. For. Res.* 10 83–86. 10.1007/BF02855532

[B19] Fan W. Lou H. Q. Yang J. L. Zheng S. J. (2016a). The roles of STOP1-like transcription factors in aluminum and proton tolerance. *Plant Signal. Behav.* 11:e1131371 10.1080/15592324.2015.1131371 PMC4883824 26689896

[B20] Fan X. Tang Z. Tan Y. Zhang Y. Luo B. Yang M. (2016b). Overexpression of a pH-sensitive nitrate transporter in rice increases crop yields. *Proc Natl. Acad. Sci. U.S.A.* 113 7118–7123. 10.1073/pnas.1525184113 27274069 PMC4932942

[B21] Fang X. Z. Tian W. H. Liu X. X. Lin X. Y. Jin C. W. Zheng S. J. (2016). Alleviation of proton toxicity by nitrate uptake specifically depends on nitrate transporter 1.1 in *Arabidopsis*. *New Phytol.* 211 149–158. 10.1111/nph.13892 26864608

[B22] Garnica-Vergara A. Barrera-Ortiz S. Muñoz-Parra E. Raya-González J. Méndez-Bravo A. Macías-Rodríguez L. (2016). The volatile 6-pentyl-2H-pyran-2-one from *Trichoderma atroviride* regulates *Arabidopsis thaliana* root morphogenesis via auxin signaling and ETHYLENE INSENSITIVE 2 functioning. *New Phytol.* 209 1496–1512. 10.1111/nph.13725 26568541

[B23] Graças J. P. Ruiz-Romero R. Figueiredo L. D. Mattiello L. Peres L. E. P. Vitorello V. A. (2016). Root growth restraint can be an acclimatory response to low pH and is associated with reduced cell mortality: a possible role of class III peroxidases and NADPH oxidases. *Plant Biol.* 18 658–668. 10.1111/plb.12443 26891589

[B24] Harman G. E. (2006). Overview of mechanisms and uses of *Trichoderma* spp. *Phytopathology.* 96 190–194. 10.1094/PHYTO-96-0190 18943924

[B25] Harman G. E. Howell C. R. Viterbo A. Chet I. Lorito M. (2004). *Trichoderma* species-opportunistic, avirulent plant symbionts. *Nat. Rev. Microbiol.* 2 43–56. 10.1038/nrmicro797 15035008

[B26] Hashem A. Allah E. F. Alqarawib A. A. Al Huqaila A. Egamberdieva D. (2014). Alleviation of abiotic salt stress in *Ochradenus baccatus* (Del.) by *Trichoderma hamatum* (Bonord.) Bainier. *J. Plant Interact.* 9 857–868. 10.1080/17429145.2014.983568

[B27] Hermosa R. Viterbo A. Chet I. Monte E. (2012). Plant-beneficial effects of *Trichoderma* and of its genes. *Microbiology* 158 17–25. 10.1099/mic.0.052274-0 21998166

[B28] Hinsinger P. Plassard C. Tang C. Jaillard B. (2003). Origins of root-mediated pH changes in the rhizosphere and their responses to environmental constraints: a review. *Plant Soil.* 248 43–59. 10.1023/A:1022371130939

[B29] Hung R. Lee S. Bennett J. W. (2013). *Arabidopsis thaliana* as a model system for testing the effects of *Trichoderma* volatile organic compounds. *Fungal Ecol.* 6 19–26. 10.1016/j.funeco.2012.09.005

[B30] Husson O. (2013). Redox potential (Eh) and pH as drivers of soil/plant/microorganism systems: a transdisciplinary overview pointing to integrative opportunities for agronomy. *Plant Soil* 362 389–417. 10.1007/s11104-012-1429-7

[B31] Inoue S. I. Takahashi K. Okumura-Noda H. Kinoshita T. (2016). Auxin influx carrier AUX1 confers acid resistance for *Arabidopsis* root elongation through the regulation of plasma membrane H+-ATPase. *Plant Cell Physiol.* 57 2194–2201. 10.1093/pcp/pcw136 27503216 PMC5434668

[B32] Iuchi S. Koyama H. Iuchi A. Kobayashi Y. Kitabayashi S. Kobayashi Y. (2007). Zinc finger protein STOP1 is critical for proton tolerance in *Arabidopsis* and corregulates a key gene in aluminum tolerance. *Proc. Natl. Acad. Sci. U.S.A.* 104 9900–9905. 10.1073/pnas.0700117104 17535918 PMC1887543

[B33] Kang T. T. Kari N. A. Maxwell B. H. Soquila J. D. (2013). The effect of various pH levels on the root growth of *Arabidopsis thaliana* seedlings. *Expedition* 2. Available at: http://ojs.library.ubc.ca/index.php/expedition/article/view/184145

[B34] Kobayashi Y. Kobayashi Y. Watanabe T. Shaff J. E. Ohta H. Kochian L. V. (2013). Molecular and physiological analysis of Al3+ and H+ rhizotoxicities at moderately acidic conditions. *Plant Physiol.* 163 180–192. 10.1104/pp.113.222893 23839867 PMC3762639

[B35] Kochian L. V. Hoekenga O. A. Piñeros M. A. (2004). How do crop plants tolerate acid soils? Mechanisms of aluminum tolerance and phosphorus efficiency. *Annu. Rev. Plant Biol.* 55 459–493. 10.1146/annurev.arplant.55.031903.141655 15377228

[B36] Kottb M. Gigolashvili T. Grobkinsky D. Piechulla B. (2015). *Trichoderma* volatiles effecting *Arabidopsis*: from inhibition to protection against phytopathogenic fungi. *Front. Microbiol.* 6:995 10.3389/fmicb.2015.00995 PMC4586454 26483761

[B37] Koyama H. Toda T. Hara T. (2001). Brief exposure to low-pH stress causes irreversible damage to the growing root in *Arabidopsis thaliana*: pectin-Ca interaction may play an important role in proton rhizotoxicity. *J. Exp. Bot.* 355 361–368. 10.1093/jexbot/52.355.361 11283181

[B38] Koyama H. Toda T. Yokota S. Zuraida D. Hara T. (1995). Effects of aluminium and pH on root growth and cell viability in *Arabidopsis thaliana* strain Landsberg in hydroponic culture. *Plant Cell Physiol.* 36 201–205. 10.1093/oxfordjournals.pcp.a078740

[B39] Lager I. Andréasson O. Dunbar T. L. Andreasson E. Escobar M. A. Rasmusson A. G. (2010). Changes in external pH rapidly alter plant gene expression and modulate auxin and elicitor responses. *Plant Cell Environ.* 33 1513–1528. 10.1111/j.1365-3040.2010.02161.x 20444216 PMC2920358

[B40] Lee S. Yap M. Behringer G. Hung R. Bennett J. W. (2016). Volatile organic compounds emitted by *Trichoderma* species mediate plant growth. *Fungal Biol. Biotechnol.* 3:7 10.1186/s40694-016-0025-7 PMC5611631 28955466

[B41] López-Bucio J. Pelagio-Flores R. Herrera-Estrella A. (2015). *Trichoderma* as biostimulant: exploiting the multilevel properties of a plant beneficial fungus. *Sci. Hort.* 196 109–123. 10.1016/j.scienta.2015.08.043

[B42] Malamy J. E. Benfey P. N. (1997). Organization and cell differentiation in lateral roots of *Arabidopsis thaliana*. *Development* 124 33–44. 9006065 10.1242/dev.124.1.33

[B43] Marschner H. (1991). Mechanisms of adaptation of plants to acid soils. *Plant Soil.* 134 1–24. 10.1007/BF00010712

[B44] Mastouri F. Bjorkman T. Harman G. E. (2010). Seed treatment with *Trichoderma harzianum* alleviates biotic, abiotic, and physiological stresses in germinating seeds and seedlings. *Phytopathology* 100 1213–1221. 10.1094/PHYTO-03-10-0091 20649416

[B45] Mastouri F. Bjorkman T. Harman G. E. (2012). *Trichoderma harzianum* enhances antioxidant defense of tomato seedlings and resistance to water deficit. *Mol. Plant Microbe Interact.* 25 1264–1271. 10.1094/MPMI-09-11-0240 22512380

[B46] Murashige T. Skoog F. (1962). A revised medium for rapid growth and bio assays with tobacco tissue cultures. *Physiol. Plant.* 15 473–497. 10.1111/j.1399-3054.1962.tb08052.x

[B47] Nieto-Jacobo M. F. Steyaert J. M. Salazar-Badillo F. B. Nguyen D. V. Rostás M. Braithwaite M. (2017). Environmental growth conditions of *Trichoderma* spp. affects indole acetic acid derivatives, volatile organic compounds, and plant growth promotion. *Front. Plant Sci.* 8:102 10.3389/fpls.2017.00102 PMC5299017 28232840

[B48] Ottenschläger I. Wolff P. Wolverton C. Bhalerao R. P. Sandberg G. Ishikawa H. (2003). Gravity-regulated differential auxin transport from columella to lateral root cap cells. *Proc. Natl. Acad. Sci. U.S.A.* 100 2987–2991. 10.1073/pnas.0437936100 12594336 PMC151453

[B49] Prusky D. Barad S. Ment D. Bi F. (2016). The pH modulation by fungal secreted molecules: a mechanism affecting pathogenicity by postharvest pathogens. *Isr. J. Plant Sci.* 63 22–30. 10.1080/07929978.2016.1151290

[B50] Rawat L. Singh Y. Shukla N. Kumar J. (2013). Salinity tolerant *Trichoderma harzianum* reinforces NaCl tolerance and reduces population dynamics of *Fusarium oxysporum* f. sp. ciceri in chickpea (*Cicer arietinum* L.) under salt stress conditions. *Arch. Phytopathology Plant Protect.* 46 1442–1467. 10.1080/03235408.2013.769316

[B51] Salas-Marina M. A. Silva-Flores M. A. Uresti-Rivera E. E. Castro-Longoria E. Herrera-Estrella A. Casas-Flores S. (2011). Colonization of *Arabidopsis* roots by *Trichoderma* atroviride promotes growth and enhances systemic disease resistance through jasmonic acid/ethylene and salicylic acid pathways. *Eur. J. Plant Pathol.* 131 15–26. 10.1007/s10658-011-9782-6

[B52] Samolski I. Rincon A. M. Pinzon L. M. Viterbo A. Monte E. (2012). The qid74 gene from *Trichoderma harzianum* has a role in root architecture and plant biofertilization. *Microbiology* 158 129–138. 10.1099/mic.0.053140-0 21948047

[B53] Sarkar A. K. Luijten M. Miyashima S. Lenhard M. Hashimoto T. Nakajima K. (2007). Conserved factors regulate signalling in *Arabidopsis thaliana* shoot and root stem cell organizers. *Nature* 446 811–814. 10.1038/nature05703 17429400

[B54] Sawaki Y. Iuchi S. Kobayashi Y. Kobayashi Y. Ikka T. Sakurai N. (2009). STOP1 regulates multiple genes that protect *Arabidopsis* from proton and aluminum toxicities. *Plant Physiol.* 150 281–294. 10.1104/pp.108.134700 19321711 PMC2675709

[B55] Shavrukov Y. Hirai Y. (2016). Good and bad protons: genetic aspects of acidity stress responses in plants. *J. Exp. Bot.* 67 15–30. 10.1093/jxb/erv437 26417020

[B56] Shoresh M. Harman G. E. Mastouri F. (2010). Induced systemic resistance and plant responses to fungal biocontrol agents. *Annu. Rev. Phytopathol.* 48 1–23. 10.1146/annurev-phyto-073009-114450 20192757

[B57] Singh A. Shahid M. Srivastava M. Pandey S. Sharma A. Kumar V. (2014). Optimal physical parameters for growth of *Trichoderma* species at varying pH, temperature and agitation. *Virol. Mycol.* 3:127 10.4172/2161-0517.1000127

[B58] Sofo A. Tataranni G. Xiloyannis C. Dichio B. Scopa A. (2012). Direct effects of *Trichoderma harzianum* strain T-22 on micropropagated shoots of GiSeLa6^®^ (*Prunus cerasus* × *Prunus canescens*) rootstock. *Environ. Exp. Bot.* 76 33–38. 10.1016/j.envexpbot.2011.10.006

[B59] Stat Soft Inc (2011). *STATISTICA, version 10.* Available at: www.statsoft.com

[B60] Trushina N. Levin M. Mukherjee P. K. Horwitz B. A. (2013). PacC and pH-dependent transcriptome of the mycotrophic fungus *Trichoderma* virens. *BMC Genomics* 14:138 10.1186/1471-2164-14-138 PMC3618310 23445374

[B61] van den Berg C. Willemsen V. Hendriks G. Weisbeek P. Scheres B. (1997). Short-range control of cell differentiation in the *Arabidopsis* root meristem. *Nature* 390 287–289. 10.1038/36856 9384380

[B62] von Uexküll H. R. Mutert E. (1995). Global extent, development and economic impact of acid soils. *Plant Soil.* 171 1–15. 10.1007/BF00009558

[B63] Yokota S. Ojima K. (1995). Physiological responses of root tips of alfalfa to low pH and aluminium stress in water culture. *Plant Soil.* 171 163–165. 10.1007/978-94-011-0221-6_46

[B64] Yuan Z. C. Liu P. Saenkham P. Kerr K. Nester E. W. (2008). Transcriptome profiling and functional analysis of *Agrobacterium tumefaciens* reveals a general conserved response to acidic conditions (pH 5.5) and a complex acid-mediated signaling involved in *Agrobacterium*-plant interactions. *J. Bacteriol.* 190 494–507. 10.1128/JB.01387-07 17993523 PMC2223696

[B65] Zeng Q. L. Huang X. H. Zhou Q. (2005). Effect of acid rain on seed germination of rice, wheat and rape. *Environ. Sci.* 26 194–197. 15859434

