# Supplementary material for: Trichoderma-Induced Acidification Is an Early Trigger for Changes in Arabidopsis Root Growth and Determines Fungal Phytostimulation
Source: Front Plant Sci. 2017 May 17;8:822. doi: 10.3389/fpls.2017.00822 (PMC5434454; doi:10.3389/fpls.2017.00822)
Supplement: Supplementary file 1 [file Presentation_1.PDF]

Supplementary Figures

Trichoderma-induced acidification is an early trigger for changes in *Arabidopsis* root growth and determines fungal phytostimulation

Authors

Ramón Pelagio-Flores[1], Saraí Esparza-Reynoso[2], Amira Garnica-Vergara[2], José López-Bucio[2], Alfredo Herrera-Estrella[1]*.

*** Correspondence:** Alfredo Herrera Estrella. E-mail: alfredo.herrera@cinvestav.mx

Supplementary Figures (S1-S7).

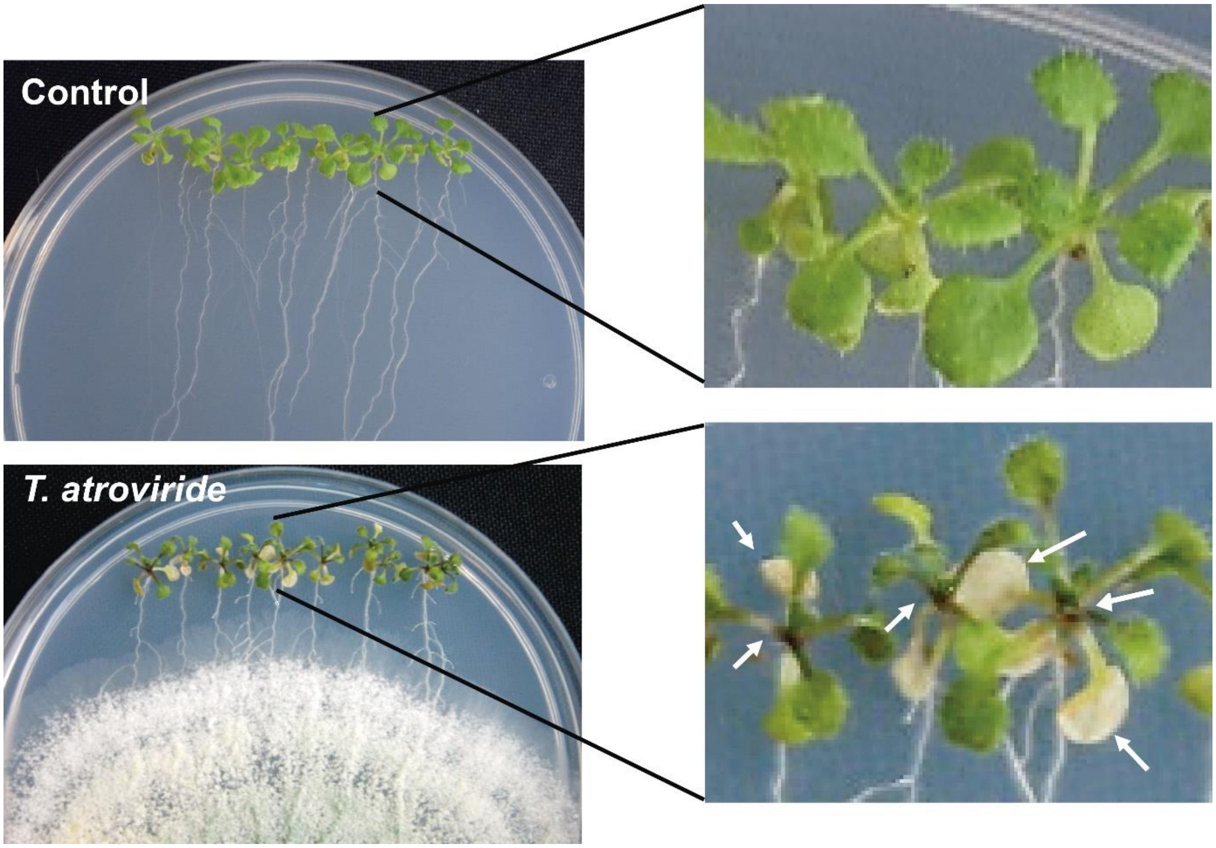

Figure S1. Late responses of *Arabidopsis* to *T. atroviride*. *Arabidopsis* seeds were germinated and growth on MS 0.2X medium. 4-day-old seedlings were inoculated with $1x10^6$ spores of *T. atroviride* in the opposite side of the plate and analyzed after 6 days of interaction. Photographs illustrate the effects of *T. atroviride* on *Arabidopsis* shoots. Arrows indicate the pigmentation or chlorosis observed in leaves.

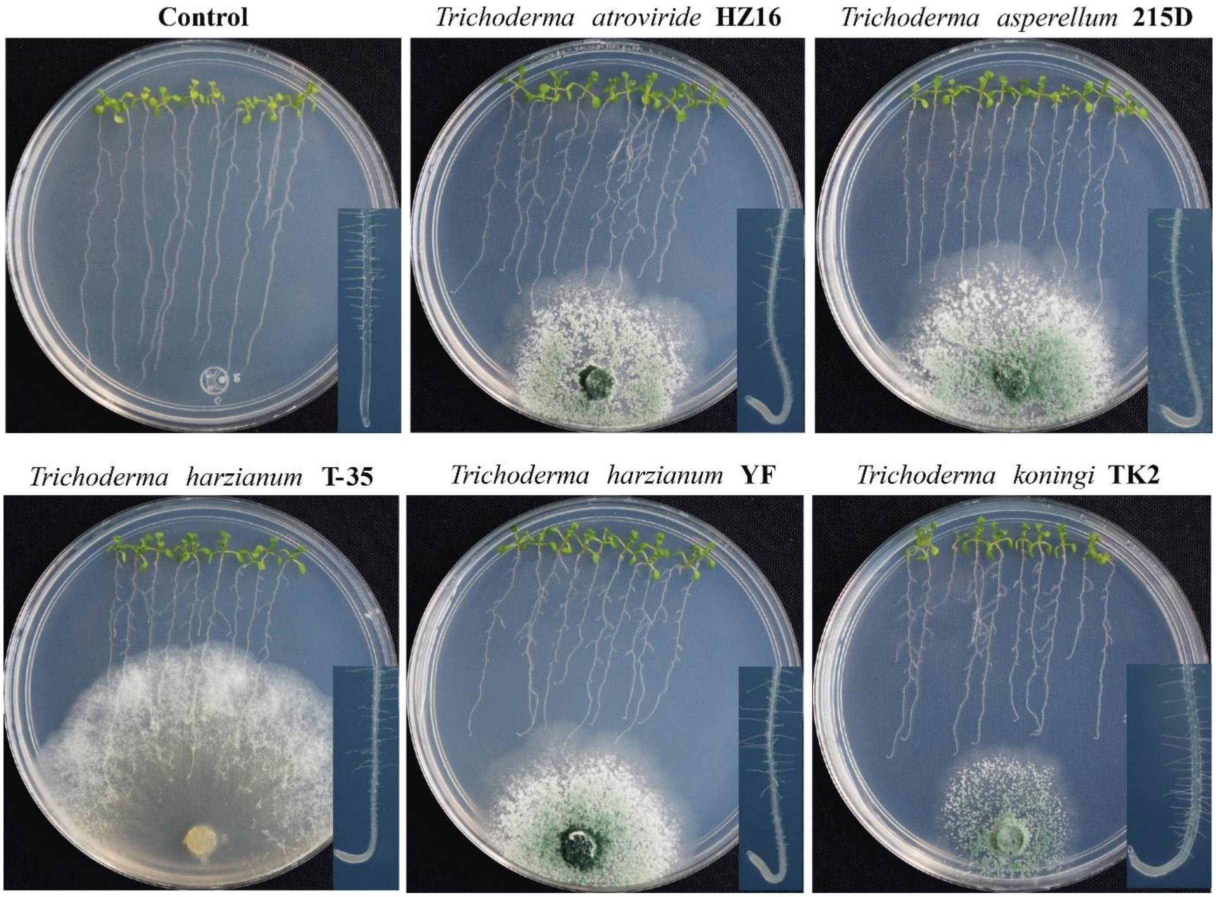

Figure S2. *Arabidopsis* root responses to different *Trichoderma* species. The same interaction conditions used in *T. atroviride* were used to evaluate the effect of the indicated *Trichoderma* species on *Arabidopsis* plant responses. Photographs illustrate the general effect after three days of interaction and the small images at the corner of each photograph show the tip root response before contact with the mycelium.

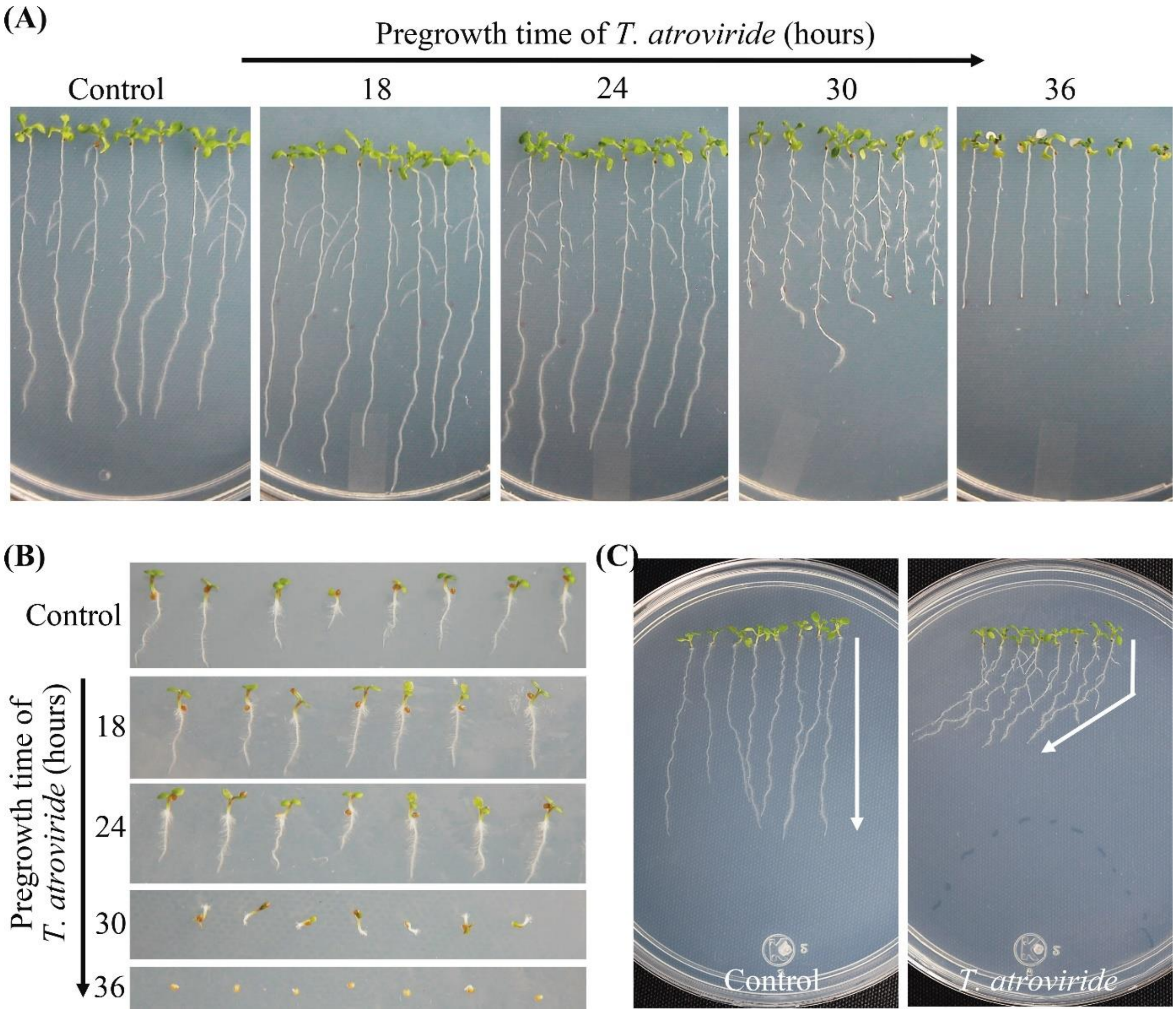

Figure S3. Gradual *Arabidopsis* responses to *T. atroviride*. (A) Effect on growth of *Arabidopsis* seedlings. (B) Effect on germination. (C) Root response. In A and B, 4d *Arabidopsis* (Col-0) seedlings were transferred or seeds germinated respectively, on MS 0.2X pH 7.0 (Control) or medium where *Trichoderma* had been pre-grown for the indicated times throughout the plate, while in C *Arabidopsis* plants were germinated and growth on medium where *Trichoderma* had been pre-grown for 48 h in a specific side of the plate and *Arabidopsis* in the opposite side.

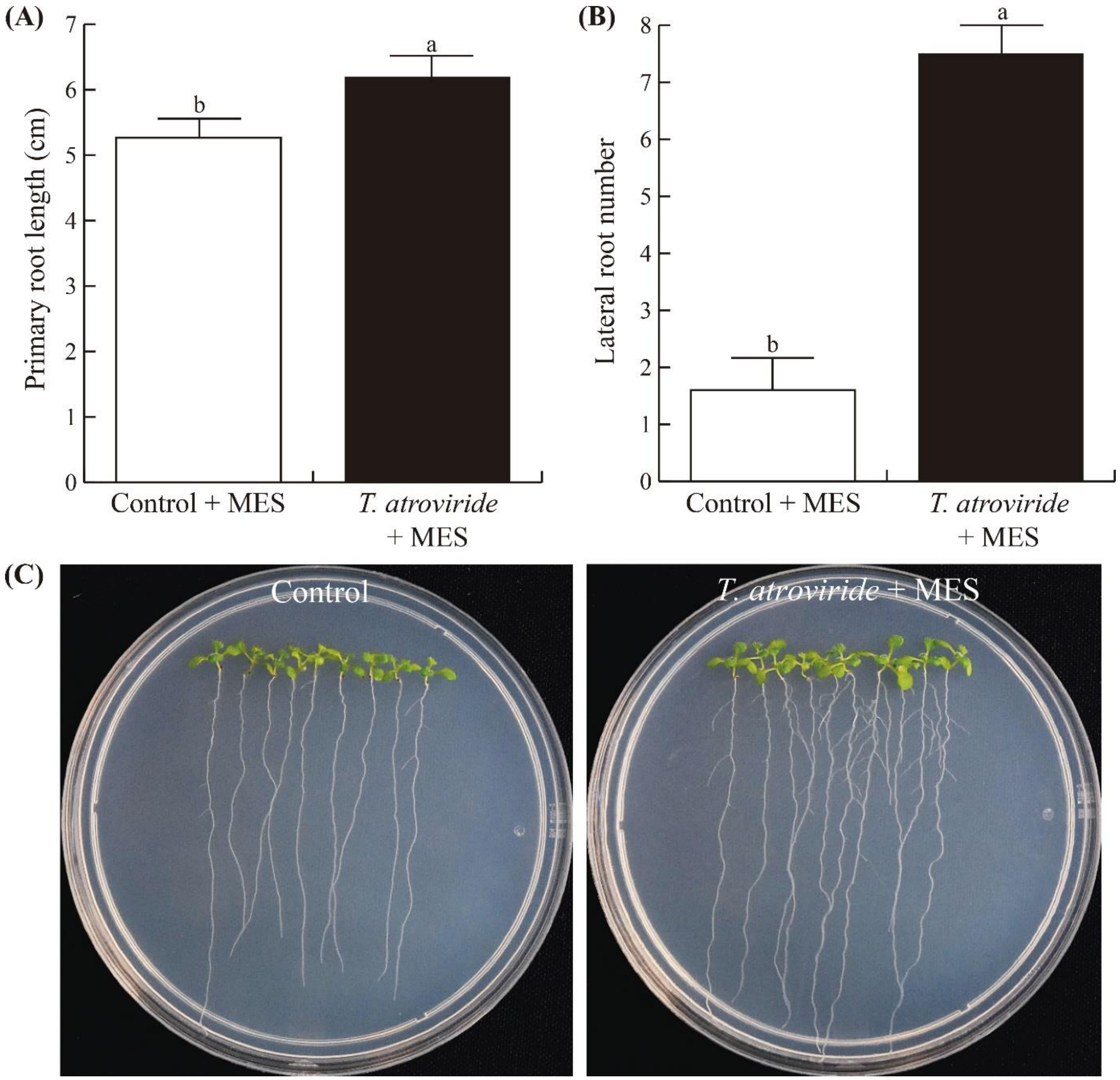

Figure S4. Effect of *T. atroviride* on *Arabidopsis* germination and their subsequent growth under buffered medium. (A) Primary root growth. (B) Lateral root number per plant. (C) Representative photographs of *Arabidopsis* seedlings under control and *Trichoderma* pregrowth conditions. As in no buffered medium *Trichoderma* was pre-grown for 36 h, retired and then seeds sown. Notice that seedlings where *Trichoderma* was pre-grown germinated and grew perfectly, being bigger than control plants and with a more branched root system.

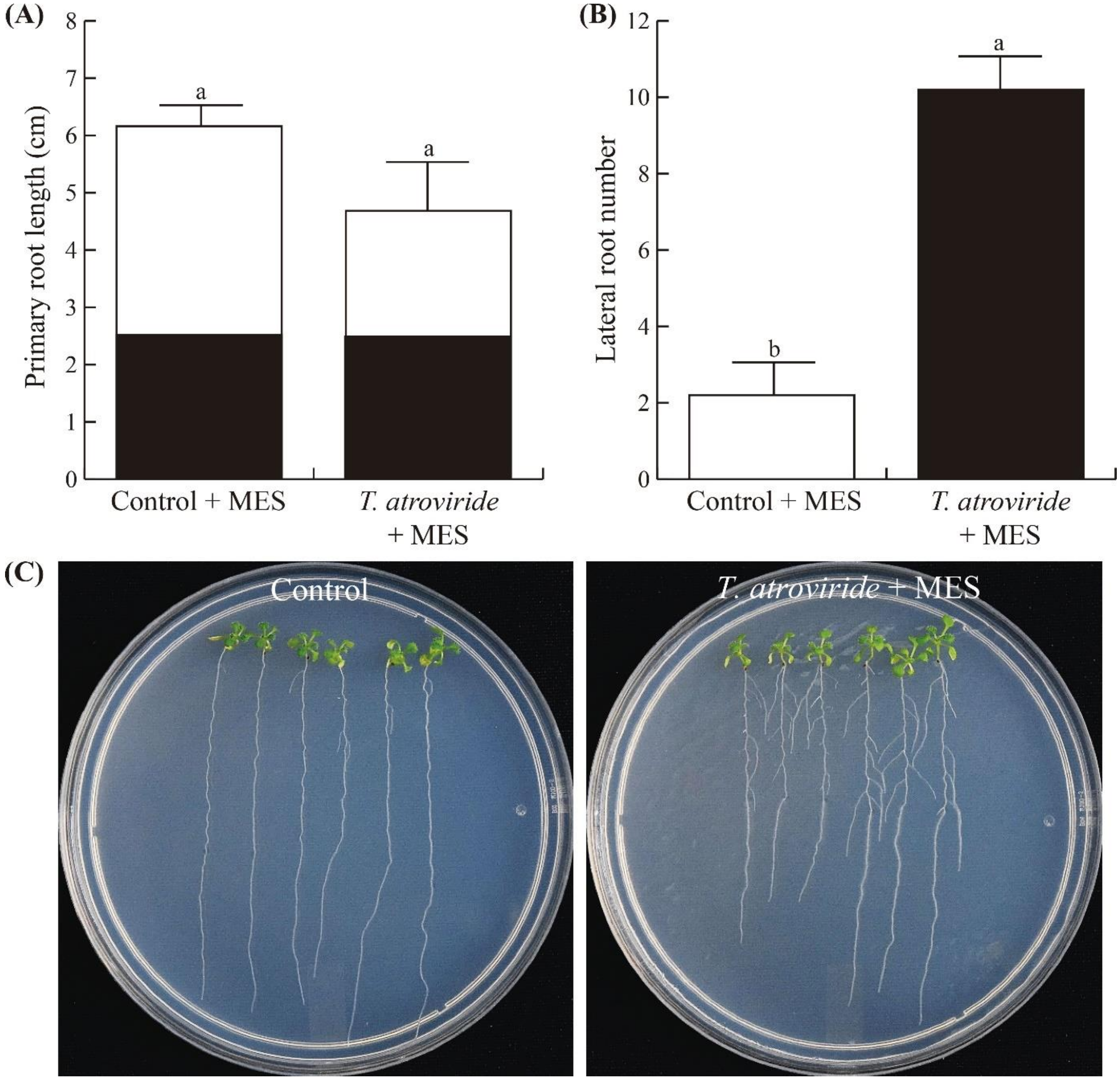

Figure S5. Effect on root growth of *T. atroviride* under buffered medium. (A) Primary root length. (B) Lateral root number per plant. (C) Representative photographs of *Arabidopsis* seedlings under control conditions and under *Trichoderma* pre-growth. *Arabidopsis* seedlings were grown on normal pH (pH 7.0) medium for 4 d and then transferred to normal (pH 7.0) or plates where *Trichoderma* had been pre-grown and analyzed 5 days later. Black bars in graph represent the root length of plants at transfer and white bars the root length 6 days after transfer.

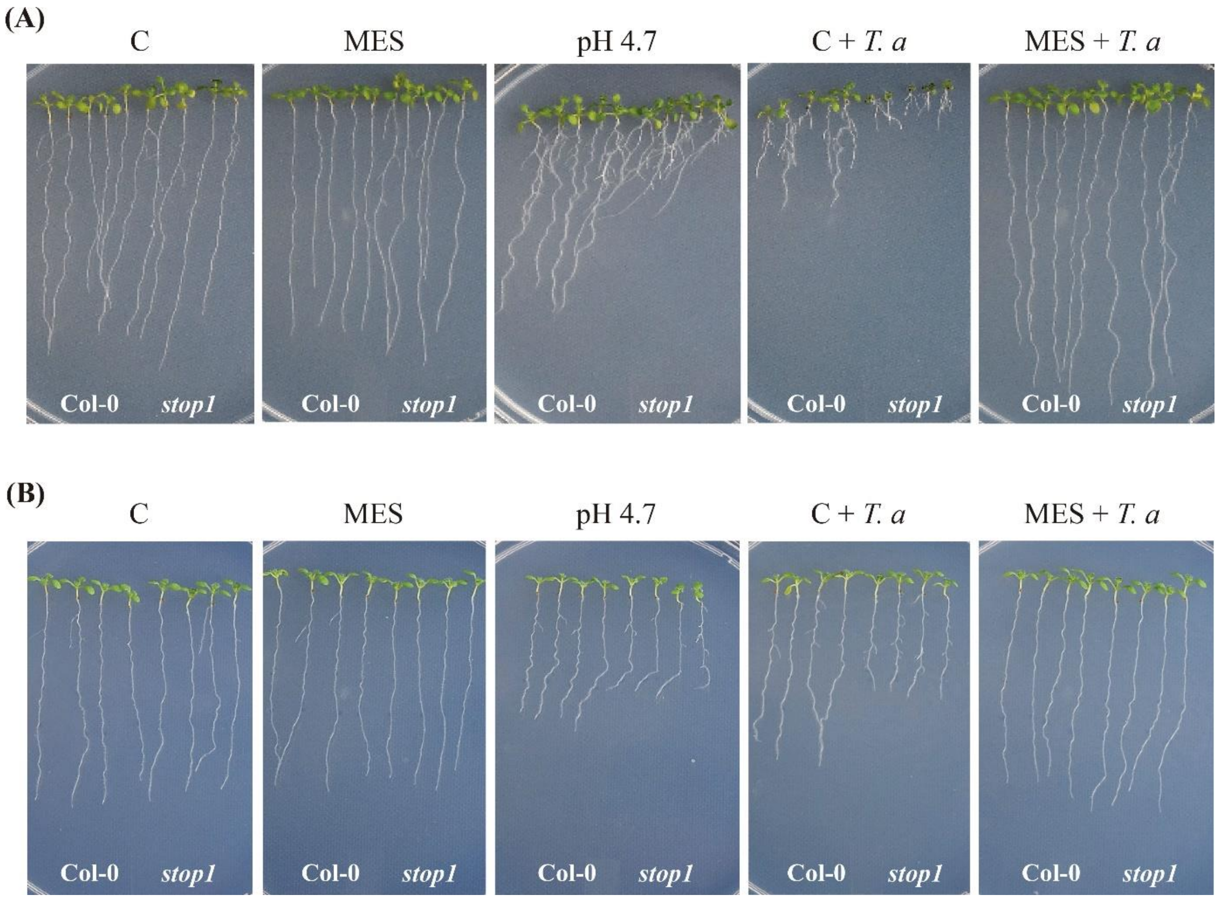

Figure S6. Phenotype of WT and *stop1* seedlings in response to *T. atroviride* in buffered or un- buffered medium. In (A) seeds were germinated and grown directly on the indicated treatments and in (B) were grown on normal pH (pH 7.0) medium for 4 d and then transferred to indicated treatments and analyzed 3 days after transfer. Notice the highest sensitivity of *stop1* compared to WT seedlings. This experiment was repeated twice with similar results.

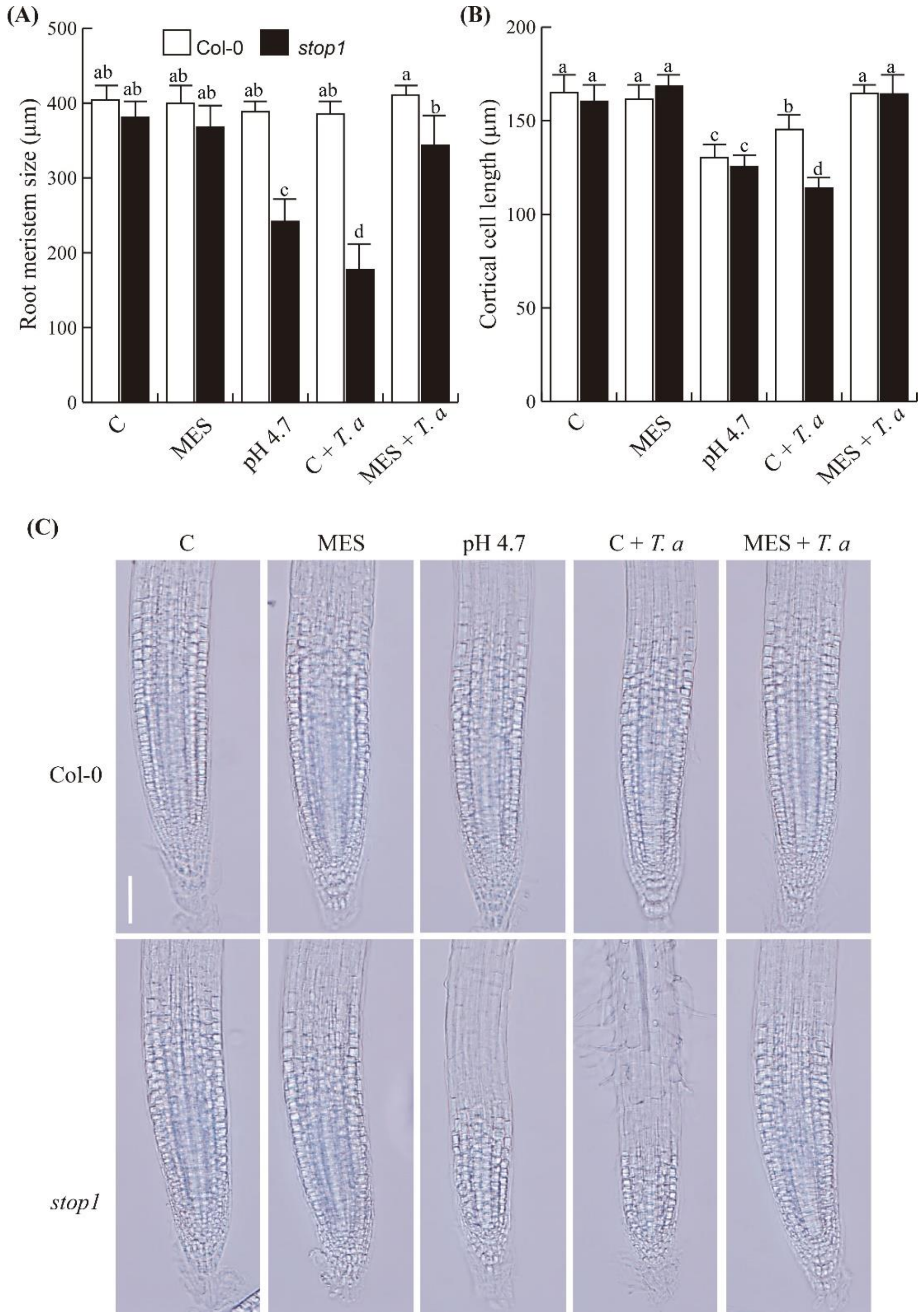

Figure S7. Effect of *Trichoderma* acidification on cell division and elongation in WT and *stop1*. (A) Root meristem length. (B) Cortical cell length. (C) Representative images of the meristems of Col-0 and *stop1* at the different treatments. WT (Col-0) and *stop1 Arabidopsis* seedlings were grown for 4 d on 0.2x MS medium and then transferred to the indicated treatments and analyzed 24 h later. The experiment was replicated twice with similar results. Different letters indicate statistical differences at $P<0.05$.
